# Antifungal activity of dendritic cell lysosomal proteins against *Cryptococcus neoformans*

**DOI:** 10.1038/s41598-021-92991-6

**Published:** 2021-06-30

**Authors:** Benjamin N. Nelson, Savannah G. Beakley, Sierra Posey, Brittney Conn, Emma Maritz, Janakiram Seshu, Karen L. Wozniak

**Affiliations:** 1 Department of Microbiology and Molecular Genetics, Oklahoma State University, 307 Life Science East, Stillwater, OK 74078 USA; 2 Department of Biology, South Texas Center for Emerging Infectious Diseases, San Antonio, TX USA

**Keywords:** Antimicrobial responses, Immunotherapy, Infection, Infectious diseases, Innate immune cells, Innate immunity, Antimicrobials, Pathogens

## Abstract

Cryptococcal meningitis is a life-threatening disease among immune compromised individuals that is caused by the opportunistic fungal pathogen *Cryptococcus neoformans*. Previous studies have shown that the fungus is phagocytosed by dendritic cells (DCs) and trafficked to the lysosome where it is killed by both oxidative and non-oxidative mechanisms. While certain molecules from the lysosome are known to kill or inhibit the growth of *C. neoformans*, the lysosome is an organelle containing many different proteins and enzymes that are designed to degrade phagocytosed material. We hypothesized that multiple lysosomal components, including cysteine proteases and antimicrobial peptides, could inhibit the growth of *C. neoformans*. Our study identified the contents of the DC lysosome and examined the anti-cryptococcal properties of different proteins found within the lysosome. Results showed several DC lysosomal proteins affected the growth of *C. neoformans *in vitro. The proteins that killed or inhibited the fungus did so in a dose-dependent manner. Furthermore, the concentration of protein needed for cryptococcal inhibition was found to be non-cytotoxic to mammalian cells. These data show that many DC lysosomal proteins have antifungal activity and have potential as immune-based therapeutics.

## Introduction

*Cryptococcus neoformans* is an encapsulated fungal pathogen that can cause pneumonia and meningitis in immune compromised individuals^1,2^. *C. neoformans* is an environmental yeast that is associated with decayed wood and pigeon droppings, and therefore, humans and animals breathe it in frequently^3,4^. Fortunately, the pathogen is typically quickly cleared from the lungs by a Th-1 type CD4^+^ T cell response before it can cause a symptomatic infection^2^. However, in cases where a person has a compromised immune system, such as reduced CD4^+^ T cells in patients with HIV/AIDS or in individuals taking immunosuppressive drugs to prevent organ transplant rejection, the fungus can cause an infection^5,6^. The prevalence between AIDS and cryptococcosis is so high that the CDC has named it an AIDS-defining illness, and the occurrence of cryptococcal meningitis is most prevalent in sub-Saharan Africa^7,8^. An analysis of the 2014 Joint UN Programme on HIV and AIDS showed that of the 223,100 yearly cases of cryptococcal meningitis, 73% occurred in sub-Saharan Africa (162,500 cases) with estimated yearly death totals of 181,100 and 135,900 worldwide and in sub-Saharan Africa, respectively^9^. While these numbers have been trending downwards in recent years, due to its high mortality rate there is still a case to be made for the World Health Organization (WHO) to name cryptococcal meningitis as a neglected tropical disease^7,10,11^. The most popular treatment programs are all based around three drugs: Amphotericin B, flucytosine, and fluconazole^12,13^. However, each is associated with certain barriers and limitations such as bioavailability, host toxicity, and emergence of resistant cryptococcal strains^14–16^.

While an adaptive T cell response is required for normal host clearance of *C. neoformans*, initial interactions with the fungus occur with innate immune cells in the lung such as dendritic cells (DCs) which are the cells responsible for antigen presentation to naive T cells^17,18^. The lungs contain a dense network of DCs, and during a cryptococcal infection, additional monocyte-derived DCs are recruited to the site of infection^19–22^. Upon encountering the fungus, DCs will phagocytose the opsonized pathogen, killing the organism in the phagolysosome^23–25^. The lysosome itself contains many different molecules (including proteases and antimicrobial peptides—AMPs) each with their own distinct role in degrading many different pathogens and/or facilitating the presentation of antigens via the MHC II pathway to the adaptive immune system^26–29^. For instance, AMPs such as cationic defensins are antifungal and the suggested mechanism of action is insertion into the fungal membrane leading to cell lysis^30–32^. Also, mice deficient in cathepsins L and S, which are cysteine proteases (these make up the majority of proteases in the lysosome), have problems with invariant chain (Ii) cleavage, which is necessary for antigen presentation^33^. Resolution of *C. neoformans* requires proper antigen presentation and activation of T cells^5,34^.

It has been previously shown that DCs (both murine bone marrow-derived DCs and human PMBC-derived DCs) can kill *C. neoformans* both in vitro and in vivo by oxidative and non-oxidative mechanisms^26,35,36^. However, it is possible for the fungus to survive and replicate inside other immune cells such as macrophages^37,38^. Furthermore, the purified contents of DC lysosomes can also kill the fungus in vitro in a dose-dependent manner^26,36^. These studies illustrate that it is indeed the components of the lysosome that act upon the pathogen. Since the lysosome is a generalized killing organelle designed to destroy many pathogens, it contains many different substances that individually may or may not have an effect on *C. neoformans*^39^. Thus far, it has been shown that one component within the DC lysosome can inhibit the growth of this yeast: cathepsin B. This cysteine protease is able to form a hole in the cell wall that results in osmotic lysis of *C. neoformans*^26^. In addition, use of enzymatic inhibitors of cathepsin B enhances the antifungal activity, suggesting that this protease may be acting in a non-enzymatic fashion^26^. Further studies are underway in our laboratory to determine cathepsin B’s mechanism of action against *C. neoformans*.

Our previous studies showed that in an acidic buffer (pH 5.5) the entire contents of the DC lysosome as well as a specific lysosomal component could kill *C. neoformans*^26,36^. The lysosome is rich in enzymes and other antimicrobial mediators^27,40^, and we hypothesized that additional lysosomal components have antifungal properties. Therefore, in the present study, the contents of the DC lysosome were investigated to identify additional anti-cryptococcal molecules that have activity in similar conditions to the DC lysosome (pH 5.5). First, the lysosome extract was fractionated by molecular weight and individual fractions were tested for antifungal activity. Next, mass spectrometry was performed on the DC lysosome extract fractions to identify all of the components present. A panel of lysosomal components was selected for their antimicrobial properties and screened against *C. neoformans *in vitro*,* and those that showed antifungal activity were further studied in dose-dependent assays. Finally, those concentrations that inhibited the growth of the fungus were assessed for cytotoxic effects against mammalian cells near physiological pH (7.5). The purpose of the current study was to identify the anticryptococcal components of the DC lysosome and to assess their potential to be used as therapeutics.

## Results

### *C. neoformans* growth is inhibited by DC-derived lysosomal fractions

**Figure 1:**
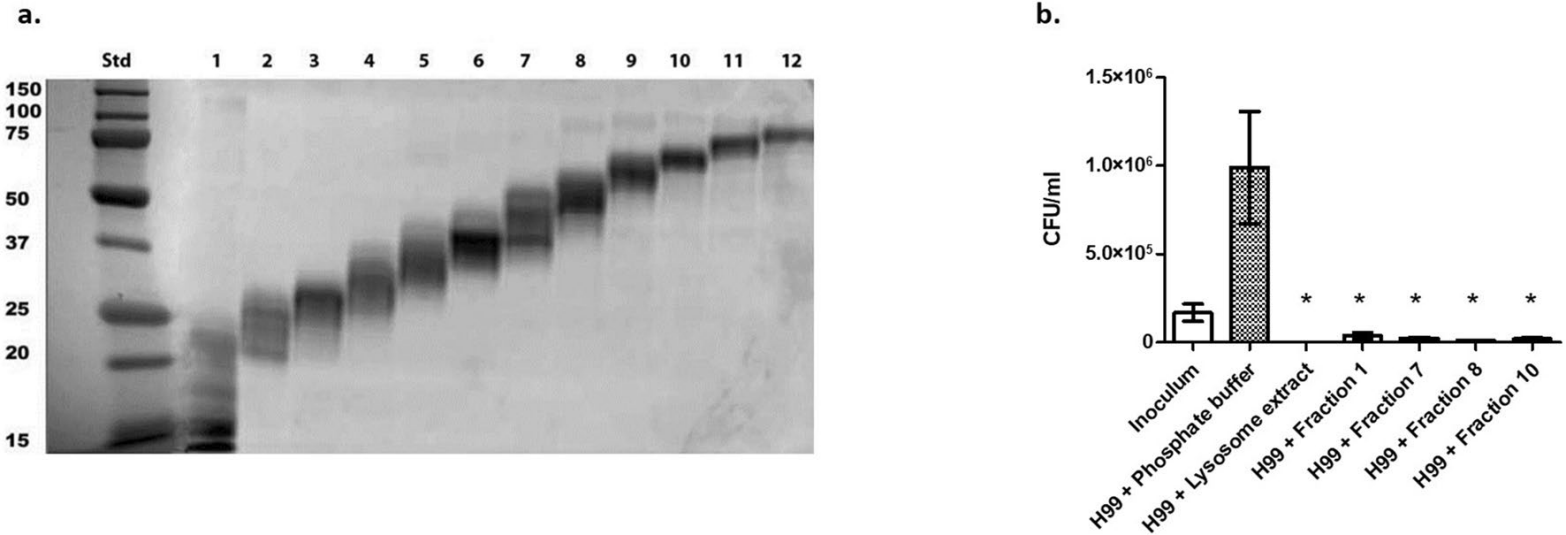
Fractions of DC Lysosomal Extract Have Anti-cryptococcal Activity. (**a**) Crude DC-derived lysosomal extracts were separated by molecular weight using a GELFrEE 8100 system into 12 different fractions and further resolved by standard SDS-PAGE. Proteins in the 12.5% gels were stained using BioSafe Coomassie Stain and visualized using a Gel Doc XR + system. (**b**) *C. neoformans* serotype A strain H99 yeast cells (1 × 10^5^ cells/ml) were incubated in phosphate buffer (pH 5.5) with whole lysosomal extract or fractions of lysosomal extract for 24 h at 37 °C, 5% CO_2_, following which CFUs in the wells were determined. Data shown are means ± standard errors of the means (SEM) of the results of three independent experiments (n = 3), with each condition performed in duplicate. Two-tailed t-tests comparing H99 inoculum to each treatment were performed with asterisks (*) indicating significantly lower CFUs of treatment compared to the H99 inoculum (fraction 8: *p* = 0.0001, all others: *p* < 0.0001).

Previous studies have shown that *C. neoformans* growth can be inhibited by DCs^21,35^ as well as by DC-derived lysosomal extract^36^. Our previous studies showed that cathepsin B, which is found in the DC lysosome, can also significantly inhibit the growth of the fungus^26^. In order to identify other proteins within the lysosome that can inhibit the growth of *C. neoformans*, DC-derived lysosomal extract was first fractionated by molecular weight, and fractions were verified by SDS-PAGE (Fig. 1a). Protein concentration was measured in each fraction, and those containing at least 50 μg/ml protein (the protein concentration that was antifungal for DC lysosomal extract^36^)—fractions 1, 7, 8, and 10—were used in anti-cryptococcal assays. *C. neoformans* strain H99 was incubated at 1 × 10^5^ cells/ml with either individual fractions in phosphate buffer (pH 5.5), total extract in phosphate buffer, or in phosphate buffer alone for 24 h at 37 °C, 5% CO_2_. All fractions that were tested (fractions 1, 7, 8, and 10) showed significant killing (*p* < 0.0001) of the cryptococcal growth when compared to the *C. neoformans* inoculum (Fig. 1b). These data show that multiple fractions of DC lysosomal extract have anti-cryptococcal activity, and therefore, there are multiple compounds within the lysosomal extract that can kill *C. neoformans* or inhibit cryptococcal growth. In order to further identify individual proteins within the lysosomal extract with anti-cryptococcal activity, the fractions were analyzed by mass spectrometry to identify the contents of each fraction. Analysis revealed over 3000 different proteins present within the lysosomal fractions (Supplementary Table S1).

### DC lysosomal proteins have anti-cryptococcal activity

**Figure 2:**
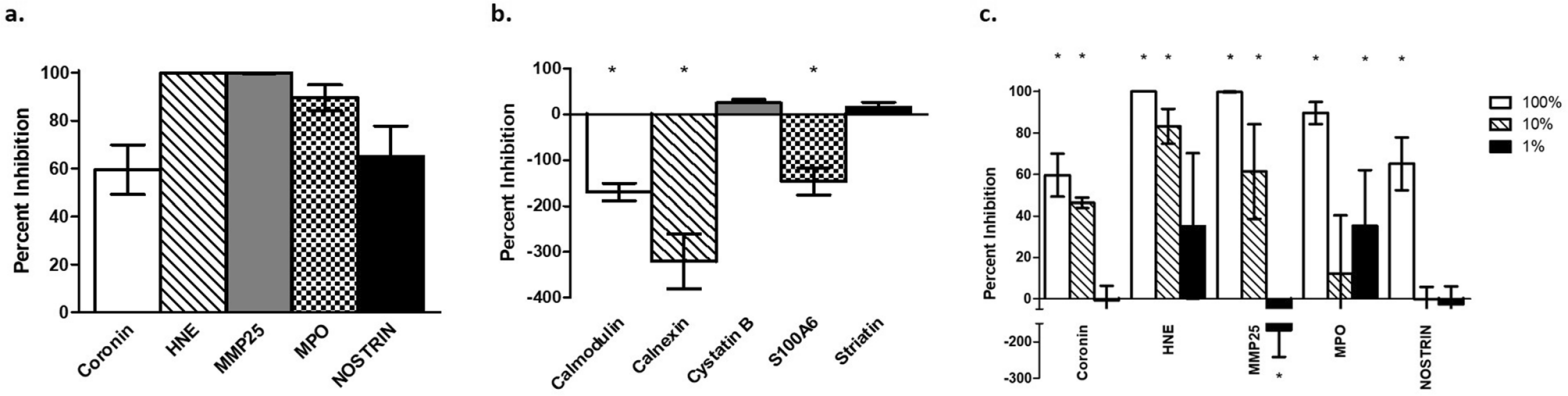
Cryptococcal Inhibition by Lysosomal Proteins. *C. neoformans* strain H99 yeast cells were grown in YPD broth for 18 h in a 30 °C shaking incubator, then washed and adjusted to a concentration of 1 × 10^5^ cells/ml. Fungi were incubated in phosphate buffer alone (pH 5.5) or in phosphate buffer with each protein for 24 h at 37 °C, 5% CO_2_, and percent inhibition defined as 100%—(test CFUs/control CFUs) × 100%. Data shown are means ± standard error of the mean (SEM) of the percent inhibition of three independent experiments (n = 3), with each condition performed in triplicate. (**a**) Lysosomal proteins that showed significant cryptococcal inhibition (*) compared to H99 in phosphate buffer alone as indicated by two-tailed t-tests (*p* < 0.0001 for all). (**b**) Lysosomal proteins that showed no cryptococcal inhibition or significantly enhanced growth (*) by two-tailed t-tests when compared to H99 in phosphate buffer alone (*p* = 0.0034 for S100A6, *p* < 0.0001 for calmodulin and calnexin)*.* (**c**) Reduced concentrations of select lysosomal proteins with white bars, striped bars, and black bars indicating protein concentration at 100%, 10%, and 1%, respectively. Two-tailed t-test comparing fungal growth with protein against growth in phosphate buffer alone confirmed significant inhibition (*) at 10% for coronin (*p* < 0.0001), HNE (*p* < 0.0001), and MMP25 (*p* = 0.0008). MPO showed significant inhibition at 1% (p = 0.0213) but not 10% while NOSTRIN was not able to reduce growth at any of the lower concentrations. At 1% original concentration, MMP25 significantly (*p* = 0.0010) increased cryptococcal growth.

In order to determine the anti-cryptococcal potential of DC lysosomal proteins, ten different lysosomal proteins were tested for anti-cryptococcal activity. From the > 3000 proteins identified by mass spectrometry, ten test proteins were selected based on potential antimicrobial activity, such as those that are known antimicrobial peptides or cysteine proteases^41–44^. Concentrations of individual proteins used in our anti-cryptococcal assay were based upon either prior published concentrations^45–48^ or were based on our previous studies with cathepsin B^26,45–48^, where concentrations of 50 and 10 µg/ml were used. Specific protein concentrations used in this study are provided in the methods section. *C. neoformans* strain H99 was incubated at 1 × 10^5^ cells/ml in a 50 μl volume separately with each protein in sterile 0.1 mM phosphate buffer, pH 5.5 (phosphate buffer) or in phosphate buffer alone for 24 h at 37 °C, 5% CO_2_, followed by diluting then plating on YPD agar to quantify CFUs. Percent cryptococcal inhibition was defined as CFUs of the experimental condition (*C. neoformans* incubated with test protein) divided by the CFUs of the control condition (*C. neoformans* incubated with phosphate buffer alone). We observed that incubation of *C. neoformans* with five of the lysosomal proteins (Coronin, HNE, MMP25, MPO, NOSTRIN) led to a significant (*p* < 0.0001) reduction of CFUs when compared to the *C. neoformans* grown in phosphate buffer alone which is shown as a high percent inhibition (Fig. 2a), while the remaining five proteins either did not inhibit fungal growth with low percent inhibition (cystatin B, striatin) or significantly enhanced fungal growth (calmodulin, *p* < 0.0001; calnexin, *p* < 0.0001; S100A6, *p* = 0.0034), as shown by negative percent inhibition (Fig. 2b).

### Cryptococcal inhibition is dose-dependent

To further elucidate the effective anti-cryptococcal concentration of each lysosomal protein, similar assays with reduced protein concentration were conducted on the five proteins that showed cryptococcal inhibition. Each of the five proteins were diluted to 10% and 1% of original concentration and incubated under the same conditions. Of those tested (coronin, HNE, MMP25, MPO, and NOSTRIN), only coronin (*p* < 0.0001), HNE (*p* < 0.0001), and MMP25 (*p* = 0.0008) significantly inhibited cryptococcal growth at the 10% protein concentration, but did not inhibit growth at the 1% concentration (Fig. 2c). Surprisingly, MMP25 significantly enhanced fungal growth (*p* = 0.0010) at 1% of the original concentration when compared to growth in phosphate buffer alone. While MPO was not able to reduce the growth of H99 at 10% concentration, when tested at 1%, it did have the capacity to significantly inhibit (*p* = 0.0213) the growth of the fungus. NOSTRIN was not effective at either of the reduced concentrations.

### Minimum inhibitory concentration (MIC) of anti-cryptococcal lysosomal proteins

Next, in order to verify that the concentrations used for the previous experiments were similar to the minimum inhibitory concentrations (MIC), the five anti-cryptococcal lysosomal proteins were evaluated for MICs against *C. neoformans* strain H99 yeast cells. Highest concentrations of proteins started at the same maximum value as previous assays and ranged from 50 µg/ml to 0.0488 µg/ml (for HNE and MMP25), 25 µg/ml to 0.0224 µg/ml (for coronin), 18 µg/ml to 0.0176 µg/ml (for MPO), and 2 µg/ml to 0.0020 µg/ml (for NOSTRIN). Proteins were assessed in phosphate buffer (pH 5.5) as in our antifungal assays above as well as a more physiological relevant condition in RPMI-MOPS (pH 7.0), which is the media typically used in fungal MIC assays. Assays were completed over 48 h at 35 °C as per Clinical and Laboratory Standards Institute (CLSI)^49–52^. Results for these assays are summarized in Table 1. When testing in lysosomal-like conditions (pH 5.5) HNE, MMP25, and NOSTRIN retained similar anti-cryptococcal efficacy. In addition, these three compounds also retained some ability to inhibit yeast growth at pH 7.0. MPO was unable to inhibit cryptococcal growth at the highest tested concentration of 18 µg/ml under both conditions and did not replicate its previous effectiveness (Fig. 2a). MIC values for coronin were not able to be determined in these assays.

**Table 1:** Minimum Inhibitory Concentrations (MIC) of Anti-cryptococcal Lysosomal Proteins in Phosphate Buffer (pH 5.5) or RPMI-MOPS (pH 7.0).

	Minimum inhibitory concentration (μg/ml)	
	Phosphate Buffer (pH 5.5)	RPMI-MOPS (7.0)
Coronin	Undetermined^1^	Undetermined^1^
HNE	3.125	50
MPO	> 18^b^	> 18^b^
MMP25	0.78	6.25
NOSTRIN	0.25	2

^a^ODs of the protein were higher than yeast growth.

^b^Growth present in the highest tested concentration.

Due to the nephrotoxicity associated with high doses of Amphotericin B (AmpB), examining methods to increase efficacy and lower dosage has become an area of intense research^53–55^. One method is the use of a combination of compounds that, by themselves, display anti-cryptococcal properties, such as current guidelines that advise treating cryptococcal meningitis using combination therapy with AmpB and flucytosine^56^. Therefore, we tested our anti-cryptococcal lysosomal proteins in combination with AmpB against the fully virulent *C. neoformans* strain H99 using a checkerboard method and categorized data based on the fractional inhibitory concentration indices (FICI)^57^. While still retaining their previously stated anti-cryptococcal capacity, none of the lysosomal proteins showed any synergistic effects when used in combination with AmpB (Supplemental Table S2). Each protein/AmpB combination scored between 1.0 and 1.8 placing them comfortably within the indifference category (0.5–4.0) and provided neither antagonistic nor synergistic results.

### Time-kill kinetics of lysosomal proteins against *C. neoformans*

**Figure 3:**
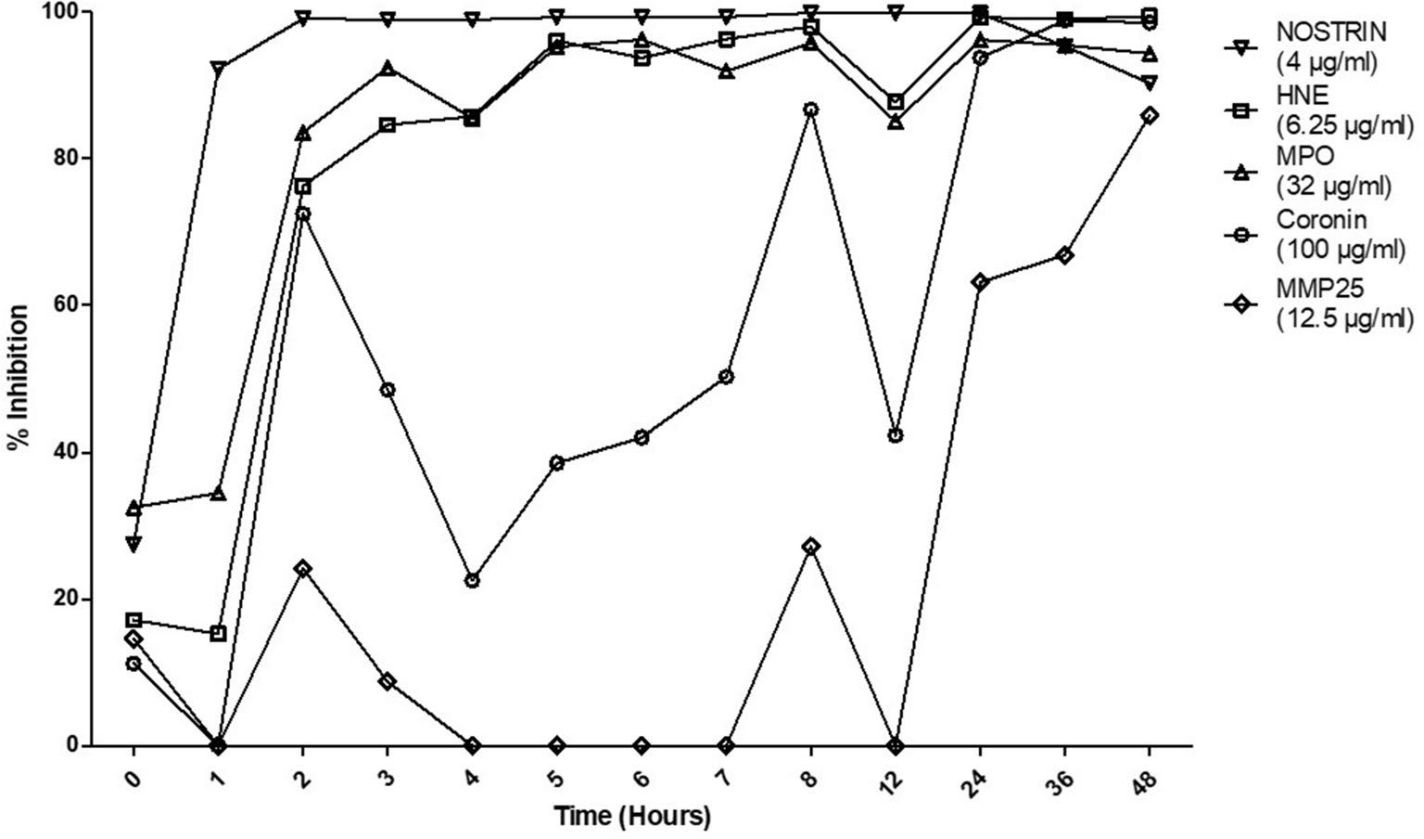
Time-kill Plots Demonstrating Cryptococcal Inhibition by Lysosomal Proteins. *C. neoformans* strain H99 yeast cells were grown in YPD broth for 18 h in a 30 °C shaking incubator, then washed and adjusted to a concentration of 1 × 10^5^ cells/ml. Fungi were incubated in phosphate buffer alone (pH 5.5) or in phosphate buffer with each protein at 2X MIC for 48 h at 35 °C. Aliquots were taken and plated for CFUs for each hour 0–8, and at 12, 24, 36, and 48 h. Percent inhibition is defined as 100%—(protein CFUs/control CFUs) × 100%. Data shown are means of two independent experiments (n = 2). Lysosomal proteins NOSTRIN, HNE, and MPO showed sustained cryptococcal inhibition by 3 h post-inoculation while coronin displayed intermediate inhibition and MMP25 exhibited no cryptococcal inhibition at the same time point. From 24–48 h all proteins inhibited cryptococcal growth.

MIC assays are useful starting points for understanding antimicrobial properties of unknown compounds, since they have been standardized by various laboratories and agencies. The time-kill assay studies the kinetic rates of inhibition over a period of time rather than examining only the end result. While they give great quantitative results and the ability to explore fungicidal rates, their methods are not as widely uniform and they can become time consuming the more time points and concentration one examines^58^. For this, we used a modified version of the method proposed in a paper by Klepser *et. al* which has since been cited over 200 times since its publication^59^. Cryptococcal cells were incubated with 2X MIC values (listed in Table 1 or 2X Table 1 or 2X highest concentration tested if none could be determined) in an acidic phosphate buffer (pH 5.5) for 48 h. As conditions change within the test chamber, properties of the proteins may also be altered, therefore, pH was monitored throughout the study, but we did not observe any changes in pH over the course of the study. We measured the percent inhibition of cryptococcal growth over time using the five anti-cryptococcal lysosomal proteins (Fig. 3). Three of the five (NOSTRIN, HNE, and MPO) displayed sustained inhibition starting from as early as three hours post inoculation. Meanwhile, at the 3 h time point, coronin and MMP25 exhibited intermediate and no inhibition, respectively. However, by 24 h, the latter two proteins showed inhibition and caught up to the other three by 48 h.

### Cytotoxicity of DC lysosomal proteins

**Figure 4:**
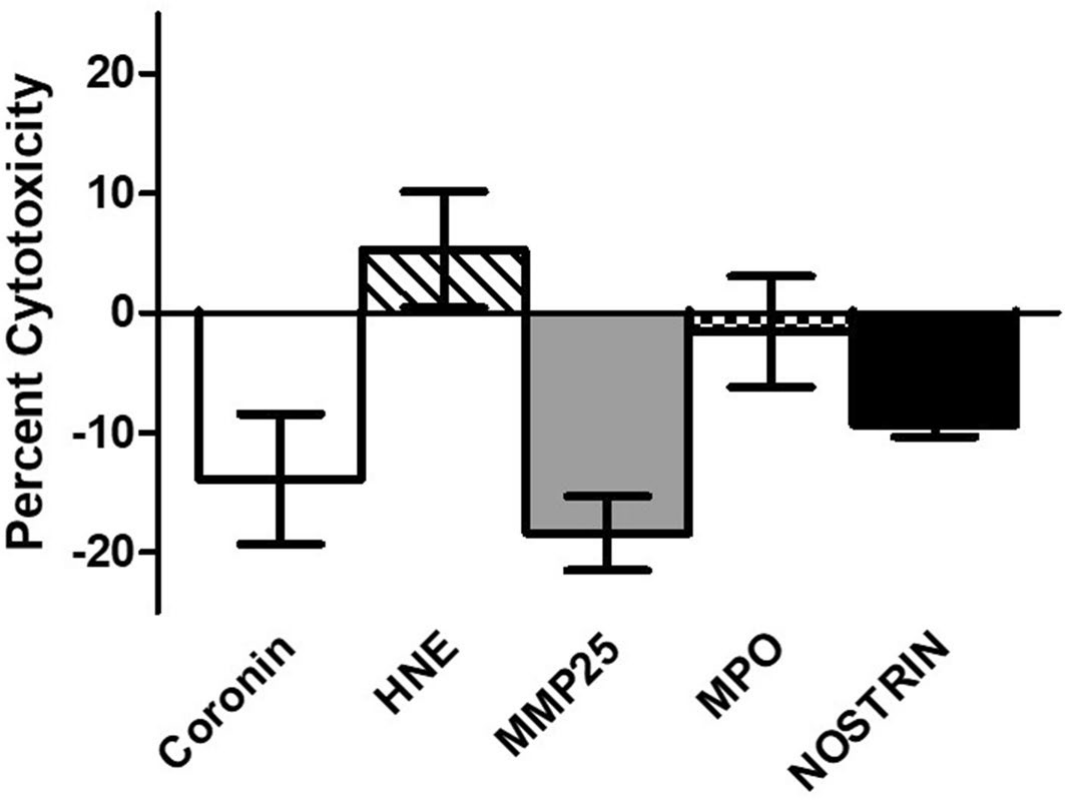
Cytotoxicity of Lysosomal Proteins. J774A.1 macrophages (5 × 10^5^ cells/ml) were incubated in cell culture media alone or cell culture media with each antifungal protein at 100% concentration for 24 h at 37 °C, 5% CO_2,_ and percent cytotoxicity was calculated as per manufacturer’s instructions (pH 7.5). Data shown are means ± standard error of the means (SEM) of the cumulative results of three independent experiments (n = 3), with each condition performed in triplicate. All proteins tested had low cytotoxicity (< 20%).

To test the relative cytotoxicity of these anti-cryptococcal lysosomal proteins on mammalian cells, a murine macrophage cell line (J774A.1) was used in conjunction with the Vybrant Cytotoxicity Assay Kit (G6PD assay) (Molecular Probes, Eugene, OR). Mammalian cells were incubated with the highest concentration of anti-cryptococcal protein (stated in methods section) in cell culture media or in cell culture media alone for 24 h at 37 °C, 5% CO_2,_ then cytotoxicity assay was performed at near physiological pH (7.5). After fluorescence was normalized to control wells (J774A.1 macrophages in cell culture media alone), percent cytotoxicity was defined as fluorescence of experimental wells (macrophages and proteins) divided by positive control wells (macrophages and cell-lysis buffer). A compound is defined as “cytotoxic” if there is a reduction of more than 30% of viable cells when compared to control wells^60^. All proteins showed low cytotoxicity (< 20%) at the highest concentration tested for cryptococcal inhibition assays (Fig. 4).

## Discussion

The primary function of the DC lysosome is to break down many different biomolecules including pathogens that may cause harm to a host in order to generate peptide fragments for antigen presentation^39,61^. This rapid degradation by the lysosome favors presentation through the MHC II pathway rather than cross presentation via MHC I^62^. Studies have shown that it can kill a wide variety of pathogens regardless of whether they are fungal, bacterial, or viral^63–67^. Previous studies have shown that *C. neoformans* traffics to the lysosomal compartment of DCs and is killed by its components^35,36^. It was also shown that contents of the DC lysosomal extract have activity against the fungus in vitro^26,36^. Furthermore, the DC lysosomal component cathepsin B can inhibit or kill the pathogen^26^. Due to the problems associated with current anti-cryptococcal drug therapies, finding new sources of treatment has become a major area of research.

To combat these ever-changing problems, most current research focuses on either discovering novel anti-fungal drugs or repurposing older drugs^68–71^. Our research focuses on identifying DC lysosomal components that are anti-cryptococcal and determining how these can be used to develop immune-based therapies to combat cryptococcosis. In accordance with our previous study on DC lysosomal proteins and their anti-cryptococcal effects, our current study has shown there are multiple DC lysosomal compounds that can inhibit and/or kill *C. neoformans* in an acidic buffer (pH 5.5) in a dose dependent manner. Three (HNE, MMP25, MPO) of the five compounds that showed anti-cryptococcal activity are known proteases that have been shown to have antimicrobial activity^72–74^. The other two are both associated with antimicrobial activity by other means: coronin is an actin binding protein that is involved with phagocytosis in leukocytes^75^ and NOSTRIN modulates the release of the oxidative stressor nitric oxide^76^. Several of these proteins still displayed inhibition at reduced concentrations which shows dose-dependent activity. Contrary to these results, however, reduced concentrations of MMP25 showed significant increase in fungal growth and may be a cause for concern if used as a therapeutic.

In order to verify that the concentrations used in these studies were in the range of minimum inhibitory concentration (MIC) concentrations, we tested the five antifungal proteins (Fig. 2) (coronin, HNE, MPO, MMP25, and NOSTRIN) in MIC assays in accordance with CLSI guidelines^49–52^. Three proteins (HNE, MMP25, and NOSTRIN) showed promising results, as not only did they recapitulate their effects in low pH, but also displayed some anti-cryptococcal activity at the higher pH, albeit at a lower efficiency. This was expected as pH changes can affect the activity of many lysosomal proteins, notably cathepsins^77^. Despite the lower activity, these results point to a future of promising therapeutics for systemic cryptococcosis, since some of these proteins can also be antifungal at neutral pH. Two proteins had different MIC results compared to our original lysosomal assays. However, differences in methodology between standard MIC assays (conducted in non-CO_2_ conditions and at 35 °C, as is standard MIC procedures in the field) and our antifungal assays (conducted at 37 °C, 5% CO_2_), may have contributed to these discrepancies.

Currently, there are only a few classes of antifungal compounds, all of which can become toxic to the host at high concentrations^53,54^. Despite being the drug of choice when treating cryptococcal meningitis, AmpB does have these same concerns, especially targeting the kidneys, causing nephrotoxicity. We sought ways to reduce standard treatment concentrations by investigating synergistic activity that may exist between it and our anti-cryptococcal lysosomal proteins. However, none of the compounds showed any synergy when combined with AmpB at any of concentrations tested. These results are not too surprising owing to the fact that the H99 strain, while fully virulent, is still susceptible to AmpB at low doses and other compounds may not show synergistic effects. These results may differ and show synergistic effects when testing against a cryptococcal strain that is resistant to AmpB. Furthermore, synergistic combinations tend to target both pathogenic processes and cell growth to completely inhibit a fungal infection^78^. More pre-clinical research in this area is needed as these studies were conducted in vitro. We also wanted to further understand the kinetics of these anti-cryptococcal lysosomal proteins, so a time-kill assay was conducted to evaluate growth over 48 h, the same as a standard MIC assay. Three (HNE, MPO, NOSTRIN) of the five proteins showed sustained inhibition quickly, within 3 h post-incubation. However, by the time point of our typical lysosomal assays (24 h), the remaining lysosomal proteins all showed inhibition of cryptococcal growth, which was sustained through the 48 h experiment.

Despite previous reports of antimicrobial activity against bacterial pathogens^79–82^ and/or interference with virulence pathways (such as the calcium-binding pathway^44,83^), several proteins tested in this study (calmodulin, calnexin, cystatin B, S100A6, and striatin) showed either no cryptococcal inhibition or enhanced fungal growth. We hypothesized that this enhanced growth may be attributed to secretion of nutrients or trace metals that may enhance cryptococcal growth and we are currently analyzing the contents of media in these conditions. Additional studies are also underway in our laboratory to examine the mechanism(s) by which these components enhance cryptococcal growth. Cystatin B is a cysteine protease that inhibits cathepsin B by tightly binding to it^84^. While cystatin B did not show any efficacy in our studies, previous work has shown that when cathepsin B and other inhibitors (calpain and CA-074) are used in conjunction with cathepsin B, they lead to increased inhibition *C. neoformans* growth when compared to cathepsin B treatment alone^26^. Striatin proteins have a diverse range of functions in both filamentous fungi and mammals including the signaling of motor functions and cell division^85–87^. This family and the closely related pyristriatins that have select antimicrobial activity against Gram positive bacteria and some filamentous fungi as well as yeasts^80^. When tested against our yeast, *C. neoformans*, striatin showed no change in the cryptococcal growth. The S100 series of proteins are known antimicrobial peptides and are also known as alarmins, and these proteins have a range of antimicrobial activity^88,89^. S100A8 and S100A9 form a heterodimer named calprotectin, which has anti-cryptococcal activity by the depletion of zinc from the fungus^90–92^. However, the related protein S100A6, which has similar zinc-binding potential, significantly enhanced the cryptococcal growth^93^. The final two proteins tested, calmodulin and calnexin, also significantly enhanced cryptococcal growth. Calmodulin is a calcium binding protein that activates calcineurin and is implicated in increased pathogen defense^44,94^. The membrane associated protein calnexin is also a signaling protein that functions as a chaperone but has been also associated with increased adaptive immunity^95^. DCs that express higher than normal levels of calnexin were able to induce T cell function greater than unmodified DCs^96^. It is currently unknown why these proteins had either limited or opposite effects than previously stated literature would suggest or by what means they increased cryptococcal growth.

To our knowledge, the cytotoxicity of DC lysosomal proteins has not been tested in mammalian cells. Our findings indicated that of the proteins tested, none were cytotoxic at the concentrations needed for anti-cryptococcal activity. This shows promise that these compounds may be used further in the treatment for cryptococcosis. However, the physiological relevance and bioavailability of these compounds in a live model remains to be seen. Our laboratory is currently in the process of testing these lysosomal components in a murine model of cryptococcosis. Furthermore, the complex interactions of these proteins with other proteins residing within the lysosome has not been investigated. None of these proteins are usually present alone or act upon pathogens individually, and we recognize that there are many more proteins and protein combinations that may add to the complexity of these interactions. To help us understand these problems, we are actively working on elucidating the mechanism(s) of action for these anti-cryptococcal proteins, studying interaction of multiple lysosomal proteins, and identifying the mechanism(s) of enhanced cryptococcal growth in our lab.

The present study has investigated the anti-cryptococcal activity of specific proteins of the DC lysosome. Five different fractions showed similar anti-cryptococcal activity to the entire extract. Mass spectrometry revealed many different compounds present within this extract that could potentially inhibit cryptococcal growth. Our results show that not only are there several that indeed inhibit cryptococcal growth, but these are also not cytotoxic to mammalian cells at these concentrations. From these findings, novel immune-based anti-cryptococcal treatments may be developed from immune-based proteins that could ease the burden brought on by this deadly disease.

## Methods

### Strains and media

*Cryptococcus neoformans* strain H99 (serotype A, mating type α) was recovered from 15% glycerol stocks stored at − 80 °C and were cultured for 18 h at 30 °C with shaking in yeast extract-peptone-dextrose (YPD) broth (BD Difco; Franklin Lakes, NJ) and collected by centrifugation. Organisms were washed three times with sterile phosphate-buffered saline (PBS), and viable yeast cells were quantified using trypan blue dye exclusion in a hemocytometer. Cryptococcal cells were resuspended in appropriate medium at the concentration needed for each experiment.

### Mice

Female and male BALB/c (*H-2*^*d*^) mice were purchased from the National Cancer Institute/Charles River Laboratories and were housed either at Oklahoma State University Animal Resources or at The University of Texas at San Antonio Small Animal Laboratory Vivarium. Mice were handled according to approved guidelines, authors complied with the ARRIVE guidelines, and the study and experimental design was approved by the Oklahoma State University Institutional Animal Care and Use Committee (IACUC) or the University of Texas at San Antonio IACUC.

### Generation of bone marrow-derived dendritic cells (BMDCs)

BMDC culture was performed as previously described^26,36^. Briefly, bone marrow was flushed from the femurs and tibiae of mice using sterile HBSS. Bone marrow cells were washed, counted, and plated in complete medium (RPMI-1640 supplemented with 10% FBS, 2 mM l-glutamine, 100 U penicillin/ml, 100 μg streptomycin/ml, and 50 mM 2-mercaptoethanol) supplemented with 20 ng/ml recombinant murine GM-CSF (Peprotech, Rocky Hill, NJ) at a concentration of 2 × 10^5^ cells/ml in 10 ml (2 × 10^6^ cells/plate). Cells were incubated at 37 °C, 5% CO_2_ for a total of eight days. At day 3 of incubation, 10 ml complete medium + GM-CSF was added to each plate. At day 6 of incubation, half of the medium was removed, and 10 ml fresh complete medium + GM-CSF was added. BMDCs were harvested at day 8, and DCs were purified by negative selection using anti-F4/80 microbeads (Miltenyi Biotec, Auburn, CA) (to remove contaminating macrophages) followed by positive selection using magnetically labeled anti-CD11c microbeads according to the manufacturer’s protocol (Miltenyi Biotec). Dendritic cell purity following this procedure was > 90% by flow cytometry.

### Generation of lysosomal extract from BMDCs

DC lysosomal extracts were obtained as previously described using the lysosome isolation kit (Sigma-Aldrich, St. Louis, MO)^26,36^. Briefly, DCs were lysed using 1X extraction buffer (Sigma-Aldrich) followed by homogenization with a PowerGen 700 homogenizer (Fisher Scientific, Pittsburgh, PA) using the 7 × 110 mm homogenizer tip passed through the cells 15–20 times to disrupt 75–80% of the cells. The homogenized cells were then centrifuged at 1000 × *g* for 10 min to remove intact cells and cellular debris. The first supernatant was removed and centrifuged at 20,000×*g* for 20 min to pellet lysosomes. Lysosome purity was verified by flow cytometry for lysosomal marker LAMP1 (BD Biosciences, San Jose, CA), which showed > 90% pure lysosomes from this procedure. The pellet containing lysosomes was resuspended and sonicated for 20 s at 40% power on a Model 300 VT Ultrasonic Homogenizer (BioLogics, Inc., Manassas, VA), resulting in lysosome extract (600 mg/ml).

### Fractionation and SDS-PAGE of lysosomal extract

Twelve molecular weight protein fractions of crude lysosomal extract were separated from crude lysosomal extract using GELFREE 8100 System (Expedeon, San Diego, CA). The system uses HEPES running buffer and Tris Acetate sample buffer (Expedeon), and the system was run according to manufacturer’s instructions as previously described^97^. Briefly, lysosomal extract was mixed with sample buffer and loaded into the 8% Tris–Acetate cartridge (Expedeon). Following loading of the sample, the instrument was paused for sample collection at predefined intervals. This process was repeated until all 12 fractions were collected. After the collection of fractions, standard SDS-PAGE was performed. For this, a 12.5% Precast Gel (Bio-Rad, Hercules, CA) was loaded with a Precision Protein Plus ladder (Bio-Rad) and each of the 12 samples. Gels were run for 55 min at 200 V in Tris/glycine/SDS running buffer (Bio-Rad). Protein bands in the gel were stained using BioSafe Coomassie Stain (Bio-Rad), and a Gel Doc XR + system (Bio-Rad) was used for visualization.

### Anti-cryptococcal assays with lysosomal fractions

Anti-cryptococcal assays were performed as previously described^26,36^. Briefly, *C. neoformans* was resuspended in phosphate buffer (0.1 mM potassium phosphate monobasic supplemented with 2% RPMI-1640, pH 5.5) at concentration of 1 × 10^5^ cells/ml and added to triplicate wells of a 96-well plate in a 50 μl volume. Lysosomal fractions were added to individual triplicate well sets at 50 μl volume for a final concentration of 50 μg/ml. Negative control wells included 50 μl *C. neoformans* with 50 μl of phosphate buffer to equal the volume of experimental wells. Positive control wells contained 50 μl *C. neoformans* and 50 μl DC lysosomal extract (at 50 ug/ml). Plates were incubated for 24 h at 37 °C, 5% CO_2_. Following incubation, cryptococcal cells in the plates were diluted in PBS and plated onto yeast extract-peptone-dextrose plates supplemented with 1 microgram/ml (ug) chloramphenicol (YPD agar). The plates were incubated at 30 °C for 2 days, and then CFUs were enumerated. Killing was defined as significantly reduced CFUs compared to the inoculum, and inhibition was defined as significantly reduced CFUs compared to *Cryptococcus* grown alone in phosphate buffer. Each assay was conducted in three independent experiments (n = 3), with each condition performed in duplicate.

### Identification of lysosomal proteins by HPLC–ESI–MS/MS

Peptide identifications were performed at the Institutional Mass Spectrometry Laboratory at The University of Texas Health Science Center at San Antonio as previously described^97^. Briefly, individual fractions were digested overnight at 37 °C with trypsin (Promega, sequencing grade, Madison, WI) in 40 mM NH_4_CO_3_/10% ACN. The tryptic peptides were extracted with 0.1% TFA followed by 0.1% TFA/50% ACN. The extracts were dried by vacuum centrifugation and resuspended in 0.5% TFA. Digests were analyzed by capillary HPLC–ESI–MS/MS using a Thermo Fisher LTQ linear ion trap mass spectrometer fitted with a New Objective PicoView 550 nanospray interface. On-line HPLC separation of the digests was accomplished with an Eksigent NanoLC micro HPLC: column, PicoFrit (New Objective; 75 μm id) packed to 10 cm with C18 adsorbent (Vydac; 218MS 5 μm, 300 Å); mobile phase A, 0.5% acetic acid/0.005% TFA; mobile phase B, 90% ACN/0.5% acetic acid/0.005% TFA; gradient 2 to 42% B in 30 min; flow rate, 0.4 μl/min. MS conditions were: ESI voltage, 2.9 kV; isolation window for MS/MS, 3; relative collision energy, 35%; scan strategy, survey scan followed by acquisition of data-dependent CID spectra of the seven most intense ions in the survey scan above a set threshold. Methionine oxidation and cysteine carbamidomethylation were considered as variable modifications for all searches. Scaffold 4.0 (Proteome Software, Portland, OR, USA) was used to conduct an X! Tandem subset search of the Mascot data followed by cross-correlation of the results of both searches. The Scaffold confidence levels for acceptance of peptide assignments and protein identifications were 95 and 99%, respectively. (Supplementary Table S1).

### Anti-cryptococcal assays using DC lysosomal proteins

Anti-cryptococcal assays were performed as described above. Proteins were chosen based on previously-described antimicrobial activity, cysteine protease activity (similar to cathepsin B)^26^ or calcium interference, which affects calcineurin, a known virulence factor for *C. neoformans*^44^. All proteins were derived from commercial sources and were > 95% pure. Each protein was prepared at a concentration determined by either published studies or from our previous studies with lysosomal proteins^26,45–48^. Lysosomal proteins tested included calmodulin (1 mM) (Enzo Life Sciences, Farmingdale, NY), calnexin (50 μg/ml) (Novus Biologicals, Centennial, CO), coronin 1a (CORO1A) (25 μg/ml) (LSBio, Seattle, WA), cystatin B (5 μg/ml) (R&D Systems), human neutrophil elastase (HNE) (50 μg/ml) (LSBio), matrix metalloproteinase-25 (MMP25) (50 μg/ml) (OriGene, Rockville, MD), myeloperoxidase (MPO) (18 μg/ml) (Novus Biologicals), recombinant nitric oxide synthase traffic inducer (NOSTRIN) (2 μg/ml) (Novus Biologicals), S100A6 (25 μg/ml) (Novus Biologicals), and striatin (100 μg/ml) (Novus Biologicals). Lysosomal proteins were diluted to a 2X concentration in 0.1 mM phosphate buffer, pH 5.5 (phosphate buffer) and added to wells (50 μl) with 1 × 10^5^ cells/ml *C. neoformans* strain H99 yeast cells diluted in phosphate buffer (50 μl) to equal 100 μl in each well, and plates were incubated at 37 °C, 5% CO_2_ for 24 h. After incubation, contents of wells were diluted and plated on YPD agar to quantify CFUs. Each protein was tested in three independent experiments (n = 3), with each condition performed in triplicate. Additional anti-cryptococcal assays were performed to test dose-dependent activity of the DC lysosomal proteins. For these, proteins used were diluted from working concentrations to 1:10 and 1:100 with phosphate buffer for 10% and 1% concentrations, respectively, and the anti-cryptococcal assay was conducted as described above. Each anti-fungal assay was conducted in three independent experiments (n = 3), with each condition performed in triplicate. Percent inhibition was defined as 100% (positive inhibition control) minus the experimental condition CFUs divided by the negative inhibition CFUs × 100%. Negative percent inhibition is the result of higher CFUs in the experimental compared to negative inhibition control.

### Minimum inhibitory concentration (MIC) assays of anti-cryptococcal DC lysosomal proteins

Fungal growth inhibition of anti-cryptococcal DC lysosomal proteins were performed according to Clinical Laboratory Standards Institute (CLSI) guidelines M27: Reference Method for Broth Dilution Antifungal Susceptibility Testing of Yeasts, 4th edition^49–52^. Proteins were evaluated in twofold dilutions in concentrations ranging from 50 µg/ml to 0.0488 µg/ml (for HNE and MMP25), 25 µg/ml to 0.0224 µg/ml (for coronin), 18 µg/ml to 0.0176 µg/ml (for MPO), and 2 µg/ml to 0.0020 µg/ml (for NOSTRIN). Dilutions occurred in either phosphate buffer (0.1 mM potassium phosphate monobasic supplemented with 2% RPMI-1640, pH 5.5) or RPMI-MOPS (RPMI-1640 supplemented with 34.5 g/L MOPS, pH 7.0) in a 96-well microtiter plate. MICs assay were conducted with a non-visible concentration (0.5 × 10^3^ cells/ml) of *C. neoformans* strain H99 yeast cells. Plates were incubated for 48 h at 35 °C and optical densities of 490 nm were taken on a Synergy HTX multi-mode plate reader (BioTek). MIC was defined as the lowest concentration of protein with no visible fungal growth. Each MIC assay was conducted in two independent experiments (n = 2), with each condition performed in at least duplicate.

### Checkerboard assay using AmpB in combination with lysosomal proteins against *C. neoformans*

Anti-cryptococcal activity of AmpB in combination with anti-cryptococcal lysosomal proteins was measured in vitro using a checkerboard method as previously described^57^. Assays were conducted in either phosphate buffer (pH 5.5) or RPMI-MOPS (pH 7.0) (both described above) in 100 µl total. A 96-well plate was used with rows A-G and columns 1–9 used to create the checkerboard titrations between AmpB and a lysosomal protein. Controls used were protein only (row H), AmpB only (column 10), cryptococcal growth control (column 11), and media control (column 12). Concentrations were evaluated in twofold dilutions ranging from 4 μg/ml to 0.062 μg/ml (for AmpB), 25 μg/ml to 0.098 μg/ml (for coronin), 50 μg/ml to 0.195 μg/ml (for HNE and MMP25), 72 μg/ml to 0.281 μg/ml (for MPO), and 2 μg/ml to 0.0078 μg/ml (for NOSTRIN). *C. neoformans* strain H99 (0.5 × 10^3^ cells/ml) was used to evaluate efficacy of combinations. Plates were incubated for 48 h at 35 °C and optical densities of 490 nm were taken on a Synergy HTX multi-mode plate reader (BioTek). Combinatory results were analyzed using the Fractional Inhibitory Concentration Index (FICI, a non-parametric model based on the Loewe additivity theory) whereas FICI ≤ 0.5 is synergistic, FICI 0.5–4 is indifference, and FICI ≥ 4 is antagonistic. FICIs were defined as the summation of individual FICs (FICI = FIC_Amp B_ + FIC_Protein_) with FICs being defined as the MIC in combination divided by the MIC alone (FIC = MIC_Combination_/MIC_Alone_). Off-scale MICs were considered to be the highest or lowest concentration tested in the assay. Each checkerboard assay was conducted in two independent experiments (n = 2) for each lysosomal protein and for each buffer.

### Time-kill assay

Rate of cryptococcal killing by lysosomal protein was measured using a time-kill assay as previously described^59^. *C. neoformans* strain H99 was tested at a concentration of 1 × 10^5^ cells/ml with lysosomal proteins at 2X their respective MICs (coronin: 25 μg/ml, HNE: 3.125 μg/ml, MPO: 18 μg/ml, MMP25: 0.78 μg/ml, NOSTRIN: 0.25 μg/ml) in 1000 μl of phosphate buffer (pH 5.5). Solutions were incubated for 48 h at 35 °C with 50 μl aliquots being taken at hours 0–8, 12, 24, 36, and 48 post inoculation. Aliquots were diluted and plated on YPD agar for 48 h at 30 °C to enumerate CFUs. Each time-kill assay was conducted in two independent experiments (n = 2) for each protein along with fungus only growth control. Percent inhibition was calculated as described above with any negative inhibition values displayed as 0% inhibition.

### Cytotoxicity assay

The macrophage cell line J774A.1 (TIB-67; ATCC, Manassas, VA) was used for cytotoxicity assays of the present study. J774A.1 cells were grown in cell culture medium (DMEM supplemented with 10% heat-inactivated fetal bovine serum (FBS), 10% NCTC-109, 1% non-essential amino acids, 100 U penicillin/ml, and 100 μg streptomycin/ml) in CytoOne T75 tissue culture flasks (USA Scientific, Ocala, FL) at 37 °C, 5% CO_2_. J774A.1 cells were passaged according to manufacturer’s directions. For detection of cytotoxicity of DC lysosomal proteins, the Vybrant Cytotoxicity Assay Kit (Molecular Probes, Eugene, OR) was used according to manufacturer’s instruction at near physiological pH (7.5). Briefly, J774A.1 cells were added to a 96-well plate in triplicate at a concentration of 10 × 10^5^ cells/ml in 25 μl. Lysosomal proteins were prepared similarly to the anti-cryptococcal assay except cell culture media was used for dilutions instead of phosphate buffer. Each protein was used at 25 μl per well. Controls included no-cell, untreated cells, and fully lysed cells. All wells were prepared in triplicate. Plates were incubated for 24 h at 37 °C, 5% CO_2_. After incubation, 50 μl of reaction mixture was added to all wells. Fluorescent readings were taken on a Synergy HTX multi-mode plate reader (BioTek, Winooski, VT) with filters for 530/25 (excitation) and 590/20 (emission). Each cytotoxicity assay was conducted in three independent experiments (n = 3), with each condition performed in triplicate.

### Statistical analysis

Unpaired two-tailed t-tests were used to compare CFUs using GraphPad Prism version 5.00 for Windows (GraphPad Software, San Diego, CA). Significant differences were defined as *p* < 0.05 and α = 0.05 unless noted otherwise.

## Supplementary Information

Supplementary Information 1.

Supplementary Information 2.

Supplementary Information 3.

## Data Availability

The mass spectrometry datasets generated during and/or analyzed during the current study are included in this published article (Supplementary Table S1).
