# Supplementary material for: Antifungal activity of dendritic cell lysosomal proteins against Cryptococcus neoformans
Source: Sci Rep. 2021 Jun 30;11:13619. doi: 10.1038/s41598-021-92991-6 (PMC8245489; doi:10.1038/s41598-021-92991-6)
Supplement: Supplementary file 1 — Supplementary Information 1. [file 41598_2021_92991_MOESM1_ESM.docx]

Supplementary Information

**Antifungal Activity of Dendritic Cell Lysosomal Proteins against *Cryptococcus neoformans***

Benjamin N. Nelson^1^, Savannah G. Beakley^1^, Sierra Posey^1^, Brittney Conn^1^, Emma Maritz^1^, Janakiram Seshu^2^, and Karen L. Wozniak^1*^

^1^ Department of Microbiology and Molecular Genetics, Oklahoma State University, Stillwater, OK, ^2^Department of Biology, South Texas Center for Emerging Infectious Diseases, San Antonio, TX

**Supplementary Table S1. Contents of Dendritic Cell Lysosomal Fractions.** Mass spectrometry was conducted on each of the twelve dendritic cell (DC) lysosomal fractions (see methods for description). Each identified protein is listed along with the gene name, and the number of hits for each protein in each fraction is listed under the corresponding fraction.
