# Supplementary material for: Antifungal activity of dendritic cell lysosomal proteins against Cryptococcus neoformans
Source: Sci Rep. 2021 Jun 30;11:13619. doi: 10.1038/s41598-021-92991-6 (PMC8245489; doi:10.1038/s41598-021-92991-6)
Supplement: Supplementary file 3 — Supplementary Information 3. [file 41598_2021_92991_MOESM3_ESM.docx]

**Supplementary Table S2. Fractional inhibitory concentration indices (FICI) of Amphotericin B in combination with lysosomal proteins against *C. neoformans*.**

| **Combination**  **Media (pH)** | **Amphotericin B in combination with** | | | | |
| --- | --- | --- | --- | --- | --- |
|  | **Coronin** | **HNE** | **MPO** | **MMP25** | **NOSTRIN** |
| Phosphate Buffer (5.5) | Indifference (1.28 ± 0.00) | Indifference (1.78 ± 0.50) | Indifference (1.03 ± 0.25) | Indifference (1.18 ± 0.04) | Indifference (1.01 ± 0.04) |
| RPMI-MOPS (7.0) | Indifference (1.07 ± 0.07) | Indifference (1.25 ± 0.03) | Indifference (1.50 ± 0.50) | Indifference (1.13 ± 0.15) | Indifferece (1.01 ± 0.20) |

Numbers in parenthesis are values for FICI ± SEM for two independent experiments.
